# Issue of Compliance with Use of Personal Protective Equipment among Wastewater Workers across the Southeast Region of the United States

**DOI:** 10.3390/ijerph16112009

**Published:** 2019-06-05

**Authors:** Tamara Wright, Atin Adhikari, Jingjing Yin, Robert Vogel, Stacy Smallwood, Gulzar Shah

**Affiliations:** 1 Department of Health Policy and Community Health, Jiann-Ping Hsu College of Public Health, Georgia Southern University, Statesboro, GA 30460, USA; tw00528@georgiasouthern.edu (T.W.); ssmallwood@georgiasouthern.edu (S.S.); gshah@georgiasouthern.edu (G.S.); 2 Department of Biostatistics, Epidemiology, and Environmental Health Sciences, Jiann-Ping Hsu College of Public Health, Georgia Southern University, Statesboro, GA 30460, USA; jyin@georgiasouthern.edu (J.Y.); rvogel@georgiasouthern.edu (R.V.)

**Keywords:** occupational exposures, personal protective equipment, safety training, Health Belief Model, wastewater workers

## Abstract

Wastewater workers are exposed to different occupational hazards such as chemicals, gases, viruses, and bacteria. Personal protective equipment (PPE) is a significant factor that can reduce or decrease the probability of an accident from hazardous exposures to chemicals and microbial contaminants. The purpose of this study was to examine wastewater worker’s beliefs and practices on wearing PPE through the integration of the Health Belief Model (HBM), identify the impact that management has on wastewater workers wearing PPE, and determine the predictors of PPE compliance among workers in the wastewater industry. Data was collected from 272 wastewater workers located at 33 wastewater facilities across the southeast region of the United States. Descriptive statistical analysis was conducted to present frequency distributions of participants’ knowledge and compliance with wearing PPE. Univariate and multiple linear regression models were applied to determine the association of predictors of interest with PPE compliance. Wastewater workers were knowledgeable of occupational exposures and PPE requirements at their facility. Positive predictors of PPE compliance were perceived susceptibility and perceived severity of contracting an occupational illness (*p* < 0.05). A negative association was identified between managers setting the example of wearing PPE sometimes and PPE compliance (*p* < 0.05). Utilizing perceived susceptibility and severity for safety programs and interventions may improve PPE compliance among wastewater workers.

## 1. Introduction

The wastewater industry involves disposing of and recycling wastewater to ensure the environment is free of municipal or industrial sewage. The people who remove the pollutants from domestic and industrial wastewaters are known as wastewater workers. The work environment at a wastewater treatment plant is regarded as being very dangerous due to the various occupational hazards that are associated with working in this industry [1,2]. The occupational hazards that the wastewater workers are exposed to continue to be an issue within the industry [3,4]. These occupational hazards include physical hazards (i.e., excessive noise levels, musculoskeletal injuries, burns by hot vapors, discomfort and physiological problems), chemical hazards (i.e., exposure to chlorine, ammonia, and sodium bisulfite), and biological hazards (i.e., blood-borne pathogens and vector-borne diseases) [5,6,7]. Exposure to these hazards increases the risk of wastewater workers developing an occupational illness throughout their career [3,8]. Additionally, researchers have proven that wastewater workers are at an increased risk for Hepatitis C, gastric cancer, and spinal abnormalities [8]. Other researchers who have an interest in the health of the workers in this industry have determined that there is an increased risk for airway, gastrointestinal, and central nervous symptoms [5], and there is an increase in mortality and cancer morbidity [3] among wastewater workers.

Workplace injuries and illnesses have become a common occurrence in the workforce in the United States. In 2016, 3.6 fatal work injuries per 100,000 full-time equivalent employees were reported by the Bureau of Labor Statistics, while in 2017, approximately 2.9 cases of nonfatal workplace injuries per 100 full-time equivalent workers were reported by private industry employers [7]. Additionally, the cost of work-related injuries and disease in the United States is approximately USD 250 billion [9]. It is useful to consider that exposures in the workplace are typically preventable by using engineering controls (i.e., improved ventilation) and administrative controls (i.e., mandating the use of nonhazardous chemicals or the use of personal protective equipment (PPE)) to create a barrier between the worker and the exposure [10]. The appropriate use of PPE will aid in reducing the economic impact that occupational illnesses have on the nation, as well as increasing the health and longevity of people in the workforce.

Following the hierarchy of controls outlined by the National Institute of Occupational Safety and Health (NIOSH) can reduce the risk of developing an occupational illness within this industry [11]. The hierarchy of controls are listed from most effective to least effective in the following order: elimination, substitution, engineering controls, administrative controls, and PPE. PPE is required by employers when elimination, substitution, and engineering controls do not work and when administrative controls are not feasible or do not provide adequate protection [12].

Many workers do not feel wearing PPE is essential to their health, so PPE is often overlooked and not considered a main factor when the overall site safety is assessed [13]. NIOSH reported that 20 million workers use PPE on a regular basis to protect themselves from job hazards [14]. In the wastewater industry, most companies mandate that employees wear safety glasses, steel toe boots, and nitrile gloves as their standard PPE while they are at work. The Occupational Safety and Health Administration (OSHA) does not mandate specific regulations for this industry, but the wastewater industry is expected to follow the regulations for “General Industry” (29 Code of Federal Regulations 1910) as they pertain to safety in the workplace [15]. To date, there are specific regulations for the construction, maritime, and agriculture industries. OSHA stated that construction workers engage in many activities, similar to the same activities that wastewater workers engage in, that may expose them to serious hazards, such as unguarded machinery, electrocutions, silica dust, and asbestos [16]. The only emphasis that has been placed on the safety of wastewater workers was after the persistence of the Ebola virus (EBOV) in wastewater that led to wastewater facilities [17].

Even though there are not specific federal guidelines to protect wastewater workers from the hazards that they encounter, wastewater workers are still expected to protect themselves from these occupational exposures by complying to the safety guidelines that have been established by their employer. Some factors that can influence or control a worker from abiding to PPE regulations include culture, economic and social factors, self-efficacy, and lack of knowledge or means [13]. Safety culture and safety climate are very important within every organization because these values determine the health and safety of the organization. Since this was a pilot study focusing on wastewater worker’s attitudes, beliefs, and practices on wearing PPE, assessing safety culture and safety climate was not the primary objective for this research. A number of research studies have been conducted to assess PPE compliance among various occupations, such as healthcare [18], carpentry [19], construction [20], and agriculture workers [21]. Nonetheless, little attention has been given to PPE compliance and occupational hazards in the wastewater industry. To date, there has not been a study conducted on PPE compliance among wastewater workers.

Several theoretical models, such as the Theory of Planned Behavior, the Transtheoretical Model, and the Health Belief Model (HBM), have been used to explain and understand the factors that influence an individual’s compliance with certain guidelines, which consequently may contribute to the adoption of certain behavior [13,22]. One of the most widely used theoretical models to examine PPE compliance is the Health Belief Model (HBM) [13,23,24]. The current research utilized the HBM to determine which barriers hinder wastewater workers from wearing PPE and exposing them to occupational hazards. The HBM has also been previously used as a theoretical framework in many studies regarding safety and compliance to explain behaviors and attitudes towards a behavior in various occupations including compliance with standard precautions (SP) [13], which are safety guidelines for healthcare workers, and pesticide safety among farmworkers [23].

The HBM is the most popular theoretical model that focuses on the beliefs of people about their decisions on a particular health behavior and includes six constructs, namely (1) perceived susceptibility to a disease or illness, such as occupational diseases and hazards; (2) perceived severity of a particular condition, such as fear of contracting an occupational disease; (3) perceived barriers, such as availability of equipment and PPE discomfort, which may prevent action; (4) perceived benefits of the recommended behavior, such as protection from diseases and infection; (5) cues to action, such as knowledge of exposure and training guidelines; and (6) self-efficacy, such as confidence that wearing PPE is beneficial [25]. The HBM proposes that for action (i.e., prevention, screening) to take place, the individual has to perceive herself or himself susceptible to a certain condition (i.e., disease), the action to prevent the condition must be beneficial enough to decrease the perception of susceptibility or severity of the condition, and the benefits from taking the action have to outweigh the barriers [25,26].

Researchers have suggested that one of the best techniques to improve health outcomes, when applied to injury and illness prevention, is through the use of health behavior theories [27,28,29]. Similar research on PPE compliance determined that the HBM was a useful theoretical framework for research involving PPE compliance, because the constructs from the HBM model predict certain behaviors and attitudes that influence the use of PPE [10]. The HBM has the potential to guide and evaluate workplace interventions so that they may be effective in reducing occupational exposures and protecting worker health. This model has not been used for investigating PPE compliance among wastewater workers until now.

The purpose of this study is to determine knowledge of PPE that is acquired by wastewater workers. This includes knowledge of injuries, accidents, and other hazardous occupational exposures in the wastewater industry. Also, this study aimed to identify wastewater worker’s beliefs and practices within the framework of the HBM, and to determine the impact that management’s attitudes, beliefs, and practices has on enforcing wearing PPE. Furthermore, this study sought to determine the predictors of PPE compliance among workers in the wastewater industry.

## 2. Materials and Methods

### 2.1. Recruitment Procedure and Participants

This cross-sectional study was conducted in 2018. A total of 125 wastewater facilities were contacted across the Southeast and 33 facilities participated. The wastewater facilities that participated in this study were located in Alabama, Georgia, Mississippi, North Carolina, South Carolina, and Tennessee. Upon approval by the Institutional Review Board of Georgia Southern University, wastewater facilities were identified through an internet search and by personal contact with wastewater facility’s managers. The manager returned a Letter of Cooperation to the lead investigator to confirm their facility’s participation in the research. For facilities in Georgia, the survey was administered in person. Facilities in the other states in the southeast (Alabama, Mississippi, North Carolina, South Carolina, and Tennessee) had the survey distributed to their email by their manager via SurveyMonkey. After agreeing to the informed consent, the participants voluntarily completed the survey.

### 2.2. Questionnaire Development

The data was collected through a four-sectio, self-reported questionnaire consisting of 45 items based on the HBM constructs (questionnaire is presented in the Supplementary Document S1). At the beginning of the survey, the research team asked each participant to indicate their job title and the wastewater certifications that they currently possess, but the identities of the participants were unknown. The questions from Section II and Section IV were obtained from another study assessing PPE compliance in military workers [10]. Questions from Section I and Section III were developed by the research team. Questions in Section I consisted of two “yes/no” questions which asked if the participant was knowledgeable of the PPE that is required at their facility and of the occupational hazards that they are exposed to at their facility. If the participant indicated “yes” to either or both of these questions, they were asked to mark each PPE that is required for them to use at their facility and each occupational exposure that they encounter in their daily operations. Questions in Section II (questions 1–39) were developed from the HBM constructs (perceived susceptibility, perceived severity, perceived benefits, perceived barriers, cues to action, and self-efficacy) to assess wastewater workers beliefs and practices on wearing PPE. Questions in Section II (questions 40–45) were asked on a 5-point scale with answers ranging thus: “strongly disagree”, “disagree”, “neither agree nor disagree”, “agree”, and “strongly agree”. Questions from Section III, titled “Management”, assessed the wastewater managers’ and supervisors’ attitudes, beliefs, and practices on ensuring their employees were compliant with the PPE guidelines at their facility. Questions from the “Management” section were asked on a 5-point scale with answers ranging thus: “never”, “rarely”, “sometimes”, “very often”, and “always”. Questions from Section IV, titled “General Employee Information”, asked the participant to circle all that apply in reference to their gender, age range, years of experience, and prior training on PPE.

The survey questions were evaluated by the research team for content validity (*n* = 5). A summative score was given for each scale. The reliability coefficient scores for each scale are as follows: knowledge of safety equipment Cronbach’s α = 0.79, knowledge of occupational exposures Cronbach’s α = 0.87, perceived susceptibility Cronbach’s α = 0.51, perceived severity Cronbach’s α = 0.67, perceived benefits Cronbach’s α = 0.69, perceived barriers Cronbach’s α = 0.71, cues to action Cronbach’s α = 0.77, self-efficacy Cronbach’s α = 0.74, and management Cronbach’s α = 0.99.

### 2.3. Statistical Analyses

All data were analyzed using the Statistical Analysis System (SAS), version 9.4, SAS Institute Inc., Cary, NC [30]. Because two sampling methods were employed for facilities in Georgia and outside of Georgia, a test for homogeneity was conducted to reasonably assume the two sampling frames could be combined. Missing data within the HBM section was inputted by randomly selected values from similar observations, utilizing the hot deck imputation method. A total of 272 participants completed the survey (yielding a 25% response rate over the 125 wastewater facilities) and this data was used for statistical analysis.

A frequency analysis was conducted using PROC SURVEYFREQ to characterize all the variables. To determine the predictors of PPE compliance, univariate and multiple linear regressions were conducted using PROC SURVEYREG. The sum of the responses to each category was calculated before the linear regression analysis was conducted. For responses within the section “Knowledge”, the responses were inputted as 1 for having the knowledge and 0 for not having the knowledge, so the summation is the total number of items of which a respondent has knowledge. Similarly, for responses within the “Safety Equipment” section, the responses are 1 for indicating the safety equipment that is required and 0 for not indicating the safety equipment that is required; the summation is the total number of items of which the respondent has indicated the safety equipment that is required at their facility. Also, for responses regarding “Experience in the Wastewater Industry”, the summative items go from 1 to 6 with 1 being “less than a year” and 6 being “over 20 years”, and the scales were summarized by the total sum relating to years of experience in the wastewater industry. For the responses within the HBM, the summative items go from 1 to 5 with 1 being “strongly disagree” and 5 being “strongly agree”. These scales were summarized by the total sum of relating Likert-scale questions. Likewise, for the responses within the management section, the summative items go from 1 to 5 with 1 being “never” and 5 being “always”, and the scales were summarized by the total sum relating to Likert-scale questions. With the sum of the Likert-scale questions being computed into a summary score, each question was weighted with equal importance. To determine the predictors of PPE compliance, univariate and multiple linear regressions were conducted using PROC SURVEYREG. For this research, the dependent variable was PPE compliance and the independent variables were predictors of PPE compliance, which included knowledge level, years of experience, constructs from the HBM, and management’s attitudes, beliefs, and practices on enforcing PPE compliance. A multiple linear regression analysis was conducted to determine which variables are predictors for PPE compliance after controlling for all other variables. Regarding the univariate linear regression model on managers and supervisor’s beliefs, attitudes, and practices on their performance to increase PPE compliance among wastewater workers (Table 6), the predictor variables are the responses to each question that were reported from the manager or supervisor. *p*-values < 0.05 were considered statistically significant.

## 3. Results

### 3.1. Description of Study Participants

Table 1 presents the demographic information, years of experience in the wastewater industry, and prior training on PPE. The participants were predominantly male (84.9%), with a small percentage that were females (11.9%). The age of the participants ranged from 18 to greater than 60 years old. Twenty-four percent of the participants had over 20 years of experience, 23.2% of the participants had 1–5 years of experience, and 3.7% of the participants had less than a year of experience in the wastewater industry. Additionally, information on the occupation and wastewater licenses held by the participants are presented in the Supplementary Materials, Tables S1 and S2. Furthermore, the majority of the participants indicated that they had taken a course on the basic safety training of PPE, and over half of the participants had taken a course on the familiarization training on how to use PPE and hazard communication training on PPE. On the contrary, under half of the participants had advanced PPE training and supervisor safety training on PPE.

### 3.2. Participant’s Knowledge of PPE and Occupational Exposures at Their Wastewater Facility

The wastewater worker’s knowledge of PPE and occupational exposures at their wastewater facility is presented in Table 2. Overall, 79.8% of wastewater workers indicated that they do wear PPE every time they are at work. The wastewater workers who responded “yes”, i.e., they do wear PPE every time at work, indicated the PPE that is mandatory for them to use at their facility. Eighty-two percent marked that they are required to wear safety shoes, with gloves (74.0%) and safety goggles (62.6%) being the next highest marked (Table 2). Wastewater workers were also asked if they knew the occupational exposures/events at their facility that could cause harm to their health or the health of their fellow workers. Ninety-six percent of the participants marked “yes”, i.e., that they were knowledgeable of the occupational hazards that they are exposed to daily at work. Some of the occupational hazards that the participants indicated they are exposed to at their wastewater facility are slips and falls, chemical hazards, abrasions, blood-borne pathogens, electrical shock, confined space, excessive noise levels, fires and explosions, and respiratory issues.

### 3.3. Perceived Susceptibility and Severity of Contracting an Occupational Illness

The participants indicated that they “strongly agreed” and “agreed” with the following statements in regards to perceived susceptibility: “I believe my chances of developing an occupational illness are great”, “I worry about getting an occupational illness”, “I feel that I have a good chance of getting an occupational illness in my career”, and “I can prevent an occupational illness” (Table 3). Moreover, the participants “strongly disagreed” and “disagreed” that small exposures to occupational hazards (i.e., chemicals, viruses, noise) will not lead them to an occupational illness.

Likewise, several wastewater workers “strongly agreed” and “agreed” that their financial security would be endangered if they developed an occupational illness, developing an occupational illness will jeopardize their career, problems from an occupational illness will last a lifetime, and that they are concerned about dying prematurely if an occupational illness is developed. Furthermore, the participants “strongly disagreed” and “disagreed” that an occupational illness will not lead to permanent changes in their health, and that they were afraid of thinking about getting an occupational illness (Table 3).

### 3.4. Perceived Benefits and Barriers of Wearing PPE

When wastewater workers were asked about the benefits of wearing PPE, they “agreed” that PPE prevents future health problems, PPE prevents exposures to the kinds of hazards that they are exposed to around the job, and that they benefit from wearing PPE. When asked about the barriers that prevented the participants from wearing PPE, the participants “agreed” and “strongly agreed” that uncomfortableness was a barrier to wearing PPE (43.7%) (Table 3).

### 3.5. Cues to Action and Self-Efficacy of PPE Compliance

Also shown in Table 3, participants indicated which cues to action are necessary to increase PPE compliance. Overall, wastewater workers “agreed” that seeing others wear PPE reminds them to wear PPE, having PPE at the location of the hazard reminds them to use it, regular and frequent education on the importance of PPE improves how often they wear it, posters throughout the facility would serve as an important reminder to wear PPE, and their supervisor sets the example on wearing PPE when being exposed to hazards.

Lastly, participants were asked to indicate their self-efficacy regarding PPE compliance. Participants indicated that they agreed that they are confident “that the PPE I use when I am exposed to hazards is the proper equipment to protect me”, “I am confident that I will remember to use PPE when I am exposed to hazards at work”, and “I am confident that I can obtain the proper PPE when I am exposed to hazards at work” (Table 3).

### 3.6. Managers’ Beliefs, Attitudes, and Practices on Enforcing PPE Compliance

Managers’ and supervisors’ beliefs, attitudes, and practices on enforcing PPE compliance is presented in Table 4. The majority of the managers and supervisors stated that they “always” ensure that PPE is available for their employees and that they “always” set the example on wearing PPE when being exposed to hazards. Respondents stated they “always” enforced wearing PPE and they are “always” aware of their employee’s compliance to wear PPE. When asked how often they provide frequent and regular education on the importance of PPE, only 30.9% indicated that they “always” provide frequent and regular education on the importance of PPE. Furthermore, when asked “how often do you threaten disciplinary action if PPE regulations are not followed”, 32.2% indicated that they will “sometimes” threaten disciplinary action, while 25.2% indicated that they “never” threaten disciplinary action when PPE regulations are not followed.

### 3.7. Predictors to Increase PPE compliance

Table 5 presents the univariate linear regression and multiple linear regression results that explain the predictor variables that can be used to increase PPE compliance among wastewater workers. The predictors evaluated in the model were knowledge level, experience in wastewater industry, perceived susceptibility, perceived severity, perceived benefits, perceived barriers, cues to action, self-efficacy, and management’s decisions on PPE compliance. Compared to the other predictor variables, only perceived susceptibility (*p* < 0.05) and perceived severity (*p* < 0.05) were positively associated with PPE compliance in the univariate linear regression model. Multiple linear regression analysis showed that only perceived severity (*p* < 0.05) had a positive association with PPE compliance after adjusting for other factors.

Table 6 presented a univariate linear regression model to determine which beliefs, attitudes, and practices of managers and supervisors can be utilized to increase PPE compliance among wastewater workers. Only participants who were supervisors or managers were asked to complete this section (*n* = 123). The results indicated that there was a negative association between PPE compliance and managers and supervisors setting the example for wearing PPE “sometimes” when being exposed to hazards (*p* < 0.05).

## 4. Discussion

This study found that most components of the HBM were not associated with PPE compliance. Participants perceiving the severity and susceptibility of contracting an occupational illness were more compliant in wearing PPE. After controlling for other factors, perceived severity was significant (*p* = 0.01). One finding from the management questions determined that managers setting the example on wearing PPE sometimes was negatively associated with PPE compliance, which indicates that setting these examples should be more recurrent for increasing PPE compliance among workers. The “always” response within the management section was used as a reference level, so a positive correlation was not indicated in these findings, but it is likely that due to the higher frequency of the “always” response indicated by the managers on certain items within the management section, the “always” response would also be positively correlated with PPE compliance.

In this present study, the PPE most often mentioned as a requirement were safety goggles, safety shoes, and gloves, which are consistent with a study on farm workers and pesticide knowledge and safety practices [31]. Also consistent with present findings, researchers reported the PPE that was provided most of the time to the construction workers in Nigeria, and this included gloves, boots, and helmet [32]. An interesting knowledge finding in the present study was that 80.8% of the wastewater workers identified respiratory issues as an occupational hazard at their facility, but only 27.8% identified respirators/facemasks as being a requirement at their facility. Findings in this study also indicate that wastewater workers are knowledgeable of mandatory PPE that is required at their wastewater facility and the occupational exposures that they encounter in their daily operations. Consistent with these findings, research has indicated that prior knowledge of safety measures increased use of PPE [33].

The perceived barriers listed in the present study were inconsistent with findings from similar studies. Studies have also assessed barriers in other occupations, such as nurses and farmers, to determine which barriers hinder them from wearing PPE or complying to SP. These barriers included: unavailability of equipment, the discomfort of gloves, interference with job skills, and being too expensive [13,31]. In the present study, only the uncomfortableness of wearing PPE was consistent with previous studies. Since several wastewater facilities are financially supported by private companies and municipalities, unavailability of equipment and equipment being expensive may not be a barrier that hinders PPE compliance among wastewater workers.

The current study found a positive correlation between perceived severity of contracting an occupational illness and compliance with PPE guidelines. The HBM proposes that as perceived severity of a certain disease increases, the likelihood of taking preventative measures to decrease the chances of developing that disease should also increase [34]. For this study, perceived severity and perceived susceptibility were positive predictors that can be used to increase PPE compliance among wastewater workers; so, as wastewater workers feel that they are at risk of developing an occupational illness, then they will be more compliant with wearing PPE. Consistent with the results of this study, researchers indicated that nurse participants in their study realized that they were at an increased risk of contracting some pathogens or diseases working in the healthcare field [35]. The participants also described that contracting a pathogen infection may lead to the development of chronic diseases, generalized infections, and possibly death. In a similar study, nurses also reported they feel their families could possibly be at risk of being infected if they do not take the necessary precautions to prevent being exposed to a blood-borne infection [13].

The results from this study show that in general, several participants agreed that cues to action would benefit them wearing PPE. In the present study, wastewater workers agreed that having posters as reminders, providing frequent education on the importance of wearing PPE, having PPE at the location of the hazard, seeing others wear PPE, and the supervisor setting the example on wearing PPE would improve their compliance with PPE regulations. A study that used the HBM to assess compliance with SP also reported that cues to action such as reminders and education on SP procedures and its importance would improve compliance to SP [13]. Additionally, research has determined that it is imperative to stress the importance of safety [19]. Research on construction workers and wearing PPE has also suggested that providing short videos, statistics, and posted reminders would be best methods to improve PPE compliance [20]. Therefore, cues to action could be an important factor that increases PPE compliance in various organizations.

Training employees on safety measures is vital in increasing their knowledge, competence, and use of safety measures at the workplace [33]. Ninety-four percent of the participants in this study received the basic safety training on PPE. This finding is consistent with previous studies (where 93.3% of the respondents received instructions on wearing PPE as the most common type of PPE training) [32] and where most workers received training on the use of PPE [19]. Findings from other research, which involved farmworkers and pesticide use, were inconsistent with the present research regarding safety training [31]. The researchers determined that 64% of the farmers had not received any safety training on how to safely handle pesticides, which put the farmers at a greater health risk.

Findings from the present research regarding management commitment towards PPE enforcement is consistent with other research. Studies also encourage supervision to ensure that PPE is comfortable, and to always check, maintain, and replace PPE to improve the practice of wearing PPE [32]. Research has also emphasized the importance of enforcing employees to comply with the use of PPE through disciplinary action, incentives, and education [19].

### 4.1. Strategies to Improve PPE Compliance among Wastewater Workers

Educational programs and safety training which utilizes the HBM and emphasizes perceived severity and perceived susceptibility could be a big factor in increasing PPE compliance among wastewater workers. A behavior change intervention is suggested to determine if it would have an impact on wastewater workers knowledge, beliefs, and practices on wearing PPE. A survey would be administered before training to determine wastewater workers’ knowledge, beliefs, and practices towards wearing PPE. The intervention would involve PPE training and addressing the barriers that hinder wastewater workers from wearing PPE. After the wastewater workers have had the training, the survey should be administered again to determine if there was a significant behavior change.

Considering the findings from this research, behavior change interventions should focus on increasing perceived susceptibility and perceived severity of contracting an occupational illness, as well as utilizing cues to action to increase PPE compliance among the workers in this industry. To increase perceived susceptibility and perceived severity, there should be increased education on occupational illnesses that can develop from working in this industry. The educational intervention should address modes of transmission, preventative measures, and negative outcomes if wastewater workers are noncompliant with PPE regulations. The intervention should also focus on using cues to action (i.e., posters, continuous reminders, training) as tools to constantly promote the use of wearing PPE. Promoting the use of wearing PPE and educating wastewater workers on the importance of wearing PPE to reduce exposures to occupational hazards may help increase the worker’s perceived benefits and self-efficacy, which will result in increasing PPE compliance among these workers. Additionally, since the seriousness of occupational illnesses in the United States is an ongoing issue, focusing more research on behavior change in the workplace is essential [10].

### 4.2. Limitations

This study has several limitations that should be noted. The data in the present study was cross-sectional, and therefore a causal relationship was not established. A prospective design should be considered for future studies to provide more definitive evidence of causality between noncompliance with PPE and contracting an occupational illness. There is little information about the long-term effects of not wearing PPE and how exposure to chemicals and blood-borne pathogens have on a worker’s health. Understanding the possible exposures and outcomes that are associated with noncompliance with PPE will allow local, state, and federal officials to understand the importance of emphasizing PPE compliance in this occupation. Another concern was that the number of managers and supervisors within this study may have been overreported by the participants (*n* = 123), because each managerial level was not distinguished. Response bias may have also been a concern due to managers and supervisors being overly anxious to participate in this research, and they may have answered the questions in a manner that is favorable to the researchers. One other concern was that the study was subject to a low response rate (25%) and social desirability bias. Researchers have also discovered that social desirability bias is common within many areas of public health research that use self-reporting surveys to examine health risk behaviors [36]. An example of social desirability bias could be that the wastewater workers indicated that they are more compliant to PPE regulations than they truly are, because they assume that is the response that the researchers are desiring. To reduce social desirability bias, it is suggested that future researchers use indirect questioning, such as the randomized response technique to increase the confidentiality of responses [37]. Another limitation was that a small convenience sample (*n* = 272) was used, so, precaution should be exercised when generalizing these results.

## 5. Conclusions

In conclusion, this was the first study to examine the association of wastewater workers and PPE compliance. This study proposes that the HBM is a successful behavior change model that determines wastewater workers’ knowledge, beliefs, and practices on wearing PPE. Wastewater workers were very cognizant of the PPE requirements and occupational exposures that are associated with working in the wastewater industry. Participants only reported uncomfortableness as a barrier to PPE compliance, but in general, they felt that wearing PPE is beneficial to their health. Additionally, the results of this study indicated that perceived susceptibility and perceived severity were the strongest predictors of PPE compliance among wastewater workers. Univariate linear regression analysis also determined that there is a negative association between PPE compliance and managers setting the example by wearing PPE some of the time when they are being exposed to hazards. This finding implies that if managers only set the example sometimes when being exposed to hazards, then PPE compliance will decrease among wastewater workers. This study also offers several theoretical and practical implications that involve utilizing the HBM for future training and interventions. Since this study is the first study in the occupational health literature to examine PPE compliance among wastewater workers, it will hopefully serve as a point of reference for researchers and policymakers to focus on the seriousness of PPE compliance in the wastewater industry before adverse health effects develop among these workers.

The findings from this study can potentially contribute to the overarching research objectives for the next decade (2016–2026) listed by the Transportation Warehouse and Utilities (TWU) council of the National Occupational Research Agenda (NORA) [38,39]. NORA’s objectives from the past decade (2006–2016) have encouraged researchers to focus on discovering solutions to emerging issues regarding worker safety and occupational health [40,41,42,43]. It is evident that it is imperative for wastewater workers to comply with PPE regulations due to the different occupational exposures that are associated with the wastewater industry. Future research on workers in the wastewater industry will hopefully bridge research gaps between occupational risk factors and prevention of adverse health outcomes among wastewater workers.

Future efforts may examine the extension of the time frame for this study to increase respondents. To better represent all wastewater workers in the Southeast Region of the United States or in the whole country, a more structured sampling plan is suggested. Future studies should also focus on utilizing random sampling, so the results can be more generalizable across the target population and various occupations. Additionally, the safety culture of wastewater facilities should also be assessed in future research to determine how the safety culture impacts wastewater workers wearing PPE. Moreover, each PPE that was associated with a certain risk was not assessed to determine the barriers as to why wastewater workers do not wear certain PPE when encountering a specific exposure type. Future research can focus on developing more specific questions to determine which barriers are associated with individual PPE use and specific occupational exposures. Furthermore, this study did not take into account the environmental factors of the wastewater workers, such as their socioeconomic status or lifestyle choices (i.e., smoking habits, alcoholism). Environmental factors can have an influence on whether wastewater workers are more compliant with wearing PPE. Future research should assess environmental factors to determine if environmental factors are associated with PPE compliance.

## Figures and Tables

**Table 1:** Characteristics of the study participants.

Variables	Frequency (*n*)	Percent (%)
Gender ^a^		
Male	230	84.9
Female	31	11.4
Other	1	0.4
Age (years) ^a^		
18–25	10	3.7
26–30	23	8.5
31–35	25	9.2
36–40	31	11.4
41–45	42	15.4
46–50	38	14.0
51–55	38	14.0
56–60	32	11.8
>60	24	8.8
Experience in the Wastewater Industry ^a^		
Less than a year	10	3.7
1–5 years	63	23.2
6–10 years	33	12.1
11–15 years	45	16.5
16–20 years	43	15.8
Over 20 years	67	24.6
Prior Training on PPE ^b^		
Familiarization Training (i.e., proper use and care of PPE)	173	63.6
Basic Safety Training (i.e., purpose of PPE)	243	94.2
Hazard Communication (i.e., chemical safety)	171	62.9
Supervisor Safety Training (i.e., keeping employees safe) ^c^	108	41.9
Advanced PPE Training (i.e., OSHA training)	123	48.1

^a^ Percentages are based on completed responses; the total responses will not equal 100%. ^b^ This question involved multiple responses; the total responses will not equal 100%. ^c^ Frequency is based on respondents who indicated that they had received this training; all managers or supervisors accounted for in this research (*n* = 123) may not have had “Supervisor Safety Training”. Abbreviations: OSHA, Occupational Safety and Health Administration; PPE, personal protective equipment.

**Table 2:** Assessment of PPE compliance and knowledge of occupational exposures at the participant’s wastewater facility (*n* = 272).

Questions from Section I	Responses (*n*)	Percent (%)
Q1: Do you wear PPE every time you are at work?		
Yes	217	79.8
No	55	20.2
Q1a: If yes, which of the following PPE are mandatory for you to use? ^a^		
Safety Shoes	203	82.5
Gloves	181	74.0
Safety Goggles	153	62.6
Hardhat	122	50.0
Earmuffs/earplugs	118	48.4
Work suit/Coveralls	75	30.5
Respirators/Facemasks	70	28.5
None of the above	13	5.3
Q2: Do you know there are occupational exposures/events at your facility that can cause injuries or harm to your health and/or the health of your fellow workers?		
Yes	262	96.3
No	10	3.8
Q2a: If yes, which are the following occupational exposures/events do you know can cause injuries or harm to your health and/or the health of your fellow workers? ^a^		
Slips and falls	253	97.3
Chemical hazards	239	91.9
Abrasions	237	91.2
Blood-borne pathogens	233	89.6
Electrical shock	230	88.5
Confined Space	229	88.1
Excessive noise levels	227	87.3
Fires and explosions	222	85.4
Respiratory issues	208	80.0
Needles	192	73.8
Vector-borne diseases	189	72.7
Discomfort and psychological problems	182	70.0
Musculoskeletal injuries	181	69.6
Chronic poisoning	170	65.4
UV radiation exposure	158	60.8
Burns by steam or hot vapors	134	51.5

^a^ This question involved multiple responses; the total responses will not equal 100%. Abbreviations: PPE, personal protective equipment; UV, ultraviolet.

**Table 3:** Percentage distribution of participant’s responses from the Health Belief Model constructs (*n* = 272).

HBM Constructs (Section II)	Strongly Agree (%)	Agree %	Neither Agree nor Disagree %	Disagree %	Strongly Disagree %
Q1–Q6: Perceived Susceptibility					
I believe my chances of developing an occupational illness are great	9.6	34.9	25.4	23.9	6.3
I worry about getting an occupational illness	7.4	31.6	25.7	26.1	9.2
I feel that I have good chance of getting an occupational illness in my career	7.4	27.2	33.5	25.4	6.6
I know people in this career field who have an occupational illness	7.0	33.8	21.3	28.7	9.6
Small exposures to occupational hazards won’t lead me to an occupational illness	1.8	11.8	19.1	46.0	21.3
I can prevent an occupational illness	24.6	43.4	19.1	9.6	3.3
Q7–Q13: Perceived Severity					
The thought of getting an occupational illness is deeply concerning	7.7	38.2	27.2	20.6	6.3
If I developed an occupational illness, my career would be in jeopardy	8.8	48.2	25.0	15.8	2.2
Problems I would experience from an occupational illness would last a lifetime	11.4	43.4	34.6	8.4	2.4
An occupational illness will not lead to permanent changes in my health	2.9	9.9	25.4	43.4	18.4
My financial security would be endangered if I developed an occupational illness	22.4	47.8	16.2	11.4	2.2
I believe I could die prematurely if I developed an occupational illness	11.8	41.2	35.3	8.8	2.9
I am afraid to even think about getting an occupational illness	6.6	17.6	34.2	32.4	9.2
Q14–Q17: Perceived Benefits					
Wearing PPE will prevent future health problems for me	19.1	52.6	16.1	8.8	3.3
PPE prevents exposure to the kinds of hazards I am around on the job	21.7	54.0	12.9	8.5	2.9
I don’t worry about getting an occupational illness when wearing PPE	7.4	31.6	26.8	28.7	5.5
I benefit by wearing PPE	32.7	56.3	7.4	2.2	1.5
Q18–Q25: Perceived Barriers					
Wearing PPE is uncomfortable	4.0	39.7	30.5	22.1	3.1
PPE interferes with my ability to do my job	0.4	19.1	34.2	39.3	7.0
PPE is not always available to me	2.7	11.4	9.6	45.2	30.9
My coworkers would make fun of me for wearing PPE	1.1	4.8	6.6	51.8	35.7
My supervisor seldom wears PPE when required	4.0	8.5	18.0	42.3	27.2
My supervisor is aware of my compliance with PPE guidelines	26.5	58.5	9.9	2.9	2.2
I would need to develop a new habit for wearing PPE, and that is difficult	1.5	10.3	16.5	50.7	21.0
Wearing PPE is just too inconvenient for me	1.1	3.7	9.6	54.8	27.2
Q26–Q34: Cues to Action					
A reminder from my supervisor everyday would be important to my wear of PPE	5.1	25.3	28.7	26.1	14.7
My supervisor checking on me would improve my wear of PPE	4.4	31.2	23.5	29.0	11.8
OSHA fining me or my employer for NOT wearing PPE is important	18.4	46.7	22.4	8.1	4.4
Posters in my facility would serve as important reminders to wear PPE	11.4	52.2	25.7	8.5	2.2
The threat of disciplinary action is an important factor in ensuring I wear PPE	14.7	39.3	21.0	20.2	4.8
Having PPE at location of hazard is critical to ensure that I wear it	21.0	54.4	14.7	8.8	1.1
If I see others wearing PPE in my area, then it reminds me to use it	14.3	58.5	15.1	10.7	1.5
Regular and frequent education on the importance of PPE improves how often I wear it	18.0	52.3	18.8	9.2	1.5
My supervisor sets the example on wearing PPE when being exposed to hazards	18.0	48.9	22.4	5.5	5.1
Q35–Q39: Self-Efficacy					
I am confident that I will remember to use PPE when I am exposed to hazards at work	30.9	59.9	6.3	2.2	0.7
I am confident I can obtain the proper PPE when I am exposed to hazards at work	33.8	59.6	3.7	2.2	0.7
I am confident that my job performance will NOT be impacted by wearing PPE	23.9	48.5	15.8	8.8	2.9
I am confident that the PPE I use when I am exposed to hazard is the proper equipment to protect my health	22.4	61.4	11.8	2.2	2.2
I am confident that after wearing the proper PPE throughout my career will prevent me from getting an occupational illness	13.2	39.7	33.1	11.8	2.2

Abbreviations: OSHA, Occupational Safety and Health Administration; PPE, personal protective equipment.

**Table 4:** Frequency of management beliefs, attitudes, and practices on enforcing PPE compliance.

Section III Questions	Response	Frequency (*n*)	Percent (%)
Q40: How often do you enforce wearing personal protective equipment?			
	Always	68	55.3
	Very often	40	32.5
	Sometimes	13	10.6
	Rarely	0	0
	Never	3	2.4
Q41: How often do you set the example on wearing personal protective equipment when being exposed to hazards?			
	Always	76	61.8
	Very often	35	28.5
	Sometimes	10	8.1
	Rarely	1	0.8
	Never	1	0.8
Q42: How often are you aware of your employee’s compliance to wear personal protective equipment?			
	Always	50	40.6
	Very often	53	43.1
	Sometimes	18	14.6
	Rarely	2	1.6
	Never	0	0
Q43: How often do you threaten disciplinary action if personal protective equipment regulations are not followed?			
	Always	18	14.6
	Very often	23	18.7
	Sometimes	32	32.2
	Rarely	19	15.4
	Never	31	25.2
Q44: How often do you ensure that personal protective equipment is available for your employees?			
	Always	85	69.1
	Very often	23	18.7
	Sometimes	12	9.8
	Rarely	1	0.8
	Never	2	1.6
Q45: How often do you provide frequent and regular education on the importance of personal protective equipment?			
	Always	38	30.9
	Very often	43	35.0
	Sometimes	34	27.6
	Rarely	3	2.4
	Never	5	4.1

Percentages based on completed responses from managers and supervisors only (*n* = 123).

**Table 5:** Univariate and multiple linear regression analysis to determine which variables may increase PPE compliance among wastewater workers.

**Univariate Linear Regression Model**				
**Predictor Variable**	**Estimate**	**Standard Error**	***p*** **-Value**	**95% CI**
Knowledge level	0.05	0.04	0.16	(−0.02, 0.13)
Experience in the wastewater industry	−0.14	0.13	0.30	(−0.41, 0.14)
Perceived susceptibility	**0.16**	0.05	**0.01**	**(0.04, 0.27)**
Perceived severity	**0.20**	0.05	**0.00**	**(0.10, 0.30)**
Perceived benefits	0.06	0.08	0.48	(−0.12, 0.24)
Perceived barriers	−0.03	0.04	0.45	(−0.11, 0.05)
Cues to action	0.05	0.03	0.11	(−0.01, 0.11)
Self-efficacy	0.06	0.06	0.30	(−0.06, 0.19)
Management impact on PPE compliance	0.00	0.08	1.00	(−0.17, 0.17)
**Multiple Linear Regression Model**				
**Predictor Variable**	**Estimate**	**Standard Error**	***p*** **-Value**	**95% CI**
Knowledge level	0.04	0.06	0.52	(−0.08, 0.16)
Experience in the wastewater industry	−0.07	0.15	0.64	(−0.40, 0.26)
Perceived susceptibility	0.11	0.06	0.10	(−0.02, 0.24)
Perceived severity	**0.20**	**0.06**	**0.01**	**(0.06, 0.33)**
Perceived benefits	−0.02	0.16	0.92	(−0.37, 0.33)
Perceived barriers	0.02	0.04	0.62	(−0.07, 0.11)
Cues to action	−0.03	0.03	0.39	(−0.09, 0.04)
Self-efficacy	0.05	0.06	0.63	(−0.18, 0.27)
Management impact on PPE compliance	0.02	0.06	0.81	(−0.12, 0.15)

Dependent variable: PPE compliance; all independent variables are labelled “predictor variables”. **Bold** values are indicated as statistically significant (*p* < 0.05). Abbreviations: CI, confidence interval; PPE, personal protective equipment.

**Table 6:** Univariate linear regression model of managers’ and supervisors’ beliefs, attitudes, and practices on PPE compliance to determine which variables will increase PPE compliance among wastewater workers.

Predictor Variable	Responses	Estimate	Standard Error	*p*-Value	95% CI
How often do you enforce wearing PPE?	Always	-	-	-	-
	Very often	0.35	0.47	0.47	(−0.67, 1.37)
	Sometimes	−0.72	0.72	0.34	(−2.29, 0.85)
	Rarely	*	*	*	*
	Never	0.90	1.81	0.63	(−3.05, 4.85)
	Intercept	3.58	0.40	<0.0001	(2.72, 4.45)
How often do you set the example on wearing PPE?	Always	-	-	-	-
	Very often	−0.13	0.29	0.66	(−0.76, 0.50)
	**Sometimes**	**−1.81**	**0.43**	**0.00**	**(−2.73. 0.88)**
	Rarely #	1.14	0.34	0.01	(0.41, 1.87)
	Never #	3.14	1.81	<0.0001	(2.41, 3.87)
	Intercept	3.86	0.40	<0.0001	(3.13, 4.59)
How often are you aware of your employee’s compliance to PPE guidelines?	Always	-	-	-	-
	Very often	0.15	0.97	0.88	(−1.97, 2.27)
	Sometimes	0.10	1.20	0.94	(−2.52, 2.71)
	Rarely	1.09	2.50	0.67	(−4.35, 6.53)
	Never	*	*	*	*
	Intercept	3.59	0.64	0.00	(2.19, 4.99)
How often do you threaten disciplinary action if PPE regulations are not followed?	Always	-	-	-	-
	Very often	0.21	0.85	0.81	(−1.64, 2.05)
	Sometimes	1.13	1.09	0.32	(−1.23, 3.50)
	Rarely	0.23	0.80	0.78	(−1.52, 1.98)
	Never	−0.07	0.60	0.91	(−1.38, 1.24)
	Intercept	3.32	0.69	0.00	(1.81, 4.84)
How often do you ensure you have PPE available for your employees?	Always	-	-	-	-
	Very often	0.08	0.54	0.88	(−1.08, 1.25)
	Sometimes	−1.13	0.60	0.08	(−2.43, 0.17)
	Rarely #	3.31	0.36	<0.0001	(2.54, 4.09)
	Never #	1.81	0.36	0.00	(1.04, 2.59)
	Intercept	3.69	0.36	<0.0001	(2.91, 4.46)
How often do you provide regular and frequent education on the importance of PPE?	Always	-	-	-	-
	Very often	0.52	1.22	0.68	(−2.13, 3.18)
	Sometimes	0.34	0.90	0.71	(−1.62, 2.31)
	Rarely	1.96	1.55	0.23	(−1.42, 5.34)
	Never	−0.42	0.69	0.55	(−1.92, 1.08)
	Intercept	3.35	0.65	0.00	(1.93, 4.77)

Only participants who were supervisors or managers were asked to complete this section (*n* = 123); ^a^ ”Always” was used as the reference level—“Always” = 5 on the Likert Scale; * Indicates that this response was not selected; #Indicates the significance is inconclusive due to low frequency (*n* = 1 or *n* = 2); Statistically significant levels are in **bold** (*p*-value < 0.05). Abbreviations: CI, confidence interval; PPE, personal protective equipment.

## Supplementary Materials

The following are available online at https://www.mdpi.com/1660-4601/16/11/2009/s1, Document S1: Survey used for Research; Table S1: Occupation of Participants; Table S2: Wastewater Licenses of Participants.
