# Supplementary material for: Issue of Compliance with Use of Personal Protective Equipment among Wastewater Workers across the Southeast Region of the United States
Source: Int J Environ Res Public Health. 2019 Jun 5;16(11):2009. doi: 10.3390/ijerph16112009 (PMC6603999; doi:10.3390/ijerph16112009)
Supplement: Supplementary file 1 [file ijerph-16-02009-s001.pdf]

Document S1

SURVEY ON PPE COMPLIANCE AMONG WASTEWATER WORKERS

The purpose of this survey is to assess wastewater worker beliefs about occupational illness and the personal protective equipment they wear at work.

Examples of personal protective equipment are ear plugs and ear muffs, respirators, chemical resistant gloves, face shields, safety glasses, chemical resistant aprons, and regular overalls just to name a few.

Occupational illness can be defined as a condition that results from exposure in a workplace to a physical, chemical, or biological agent to the extent that the health of the worker is impaired. Examples of occupational illness include hearing loss from exposure to loud noise, respiratory illnesses or development of cancer from exposure to dust or chemicals and contracting viruses such as HIV and Hepatitis from handling sewage water.

The completing of the survey is voluntary and the results are anonymous. Completing or not completing the survey has no effect on the respondent's employment, and completing the survey means the subject agrees to have their responses included in the study.

Subject Identification Number: [ ]

Current Job Title: ____

Wastewater Certifications: ______________________________________

Date: _____/_____/__________
Month Day Year

GENERAL TIPS BEFORE YOU START

- **This survey will ask you mainly about your beliefs and your health.**
- **Read the whole question before making an answer.**
- **Try to answer all questions.**
- **Remember, the survey is completely anonymous.**
- **Once again, this survey is NOT an audit, and you will NOT get reprimanded for honestly answering questions regarding your beliefs and practices on wearing PPE**

SECTION I: OCCUPATIONAL HAZARDS AND PPE KNOWLEDGE

Please circle yes or no or place a check mark next to each response to each question that corresponds with your own personal beliefs and knowledge

1) Do you wear personal protective equipment every time you are at work?

Yes or No

If yes, which of the following personal protective equipment are mandatory for you to use:

Hard Hat		Work suit/coveralls	
Safety Goggles		Ear muffs/ear plugs	
Gloves		Safety Shoes (Rubber/Steel Toed	
Respirators/Face mask		None of the Above	

Other____

2) Do you know there are occupational exposures/events at your facility that can cause injuries or harm to your health and/or the health of your fellow workers?

Yes or No

If yes, which of the following occupational exposures do you know can cause injuries or harm to your health and/or the heath of your fellow workers?

Blood-borne pathogens (Hepatitis B, HIV)	
Vector Borne Diseases (From mosquitoes, ticks)	
Needles	
Abrasions (Minor Cuts, Open-wounds, Scrapes)	
Chemical hazards (Chlorine, Ammonia, Sodium Bisulfite)	
Chronic poisoning (inhalation)	
Respiratory Issues (Dust, Bio-Aerosols, Gases)	
Confined Space (Lack of Oxygen)	
Musculoskeletal injuries (Pain in the joints, muscles and ligaments from repetitive movement)	
Burns by steam or hot vapors	
Slips and Falls	
Electrical Shock	
Fires and explosions (methane, hydrogen sulfide)	
Exposure to Excessive Noise Levels	
Exposure to UV radiation	
Discomfort and psychological problems (PPE use, bad smells)	

Other____

SECTION II: HEALTH BELIEF MODEL CONSTRUCTS

Circle the number that corresponds most closely to your level of agreement with each statement.

PERCEIVED SUSCEPTIBILITY

1. I believe my chances of developing an occupational illness are great.

Strongly Disagree	*Disagree*	*Neither Agree or Disagree*	*Agree*	*Strongly Agree*
1	*2*	*3*	*4*	*5*

2. I worry about getting an occupational illness.

Strongly Disagree	*Disagree*	*Neither Agree or Disagree*	*Agree*	*Strongly Agree*
1	*2*	*3*	*4*	*5*

3. I feel that I have a good chance of getting an occupational illness in my career.

Strongly Disagree	*Disagree*	*Neither Agree or Disagree*	*Agree*	*Strongly Agree*
1	*2*	*3*	*4*	*5*

4. I know people in this career field who have an occupational illness.

Strongly Disagree	*Disagree*	*Neither Agree or Disagree*	*Agree*	*Strongly Agree*
1	*2*	*3*	*4*	*5*

5. Small exposures to chemicals, viruses or noise won't lead me to an illness.

Strongly Disagree	*Disagree*	*Neither Agree or Disagree*	*Agree*	*Strongly Agree*
1	*2*	*3*	*4*	*5*

6. I can prevent an occupational illness.

Strongly Disagree	*Disagree*	*Neither Agree or Disagree*	*Agree*	*Strongly Agree*
1	*2*	*3*	*4*	*5*

PERCEIVED SEVERITY

7. The thought of getting an occupational illness deeply concerns me.

Strongly Disagree	Disagree	Neither Agree or Disagree	Agree	Strongly Agree
1	2	3	4	5

8. If I developed an occupational illness, my career would be in jeopardy.

Strongly Disagree	Disagree	Neither Agree or Disagree	Agree	Strongly Agree
1	2	3	4	5

9. Problems I would experience from an occupational illness would last a long time.

Strongly Disagree	Disagree	Neither Agree or Disagree	Agree	Strongly Agree
1	2	3	4	5

10. An occupational illness will not lead to permanent changes in my health.

Strongly Disagree	Disagree	Neither Agree or Disagree	Agree	Strongly Agree
1	2	3	4	5

11. My financial security would be endangered if I developed an occupational illness.

Strongly Disagree	Disagree	Neither Agree or Disagree	Agree	Strongly Agree
1	2	3	4	5

12. I believe I could die prematurely if I developed an occupational illness.

Strongly Disagree	Disagree	Neither Agree or Disagree	Agree	Strongly Agree
1	2	3	4	5

13. I am afraid to even think about getting an occupational illness.

Strongly Disagree	Disagree	Neither Agree or Disagree	Agree	Strongly Agree
1	2	3	4	5

PERCEIVED BENEFITS

14. Wearing personal protective equipment will prevent future health problems for me.

Strongly Disagree	*Disagree*	*Neither Agree or Disagree*	*Agree*	*Strongly Agree*
1	*2*	*3*	*4*	*5*

15. Personal protective equipment prevents exposure to the kinds of hazards I am around on the job.

Strongly Disagree	*Disagree*	*Neither Agree or Disagree*	*Agree*	*Strongly Agree*
1	*2*	*3*	*4*	*5*

16. I don't worry about getting an occupational illness when I use personal protective equipment.

Strongly Disagree	*Disagree*	*Neither Agree or Disagree*	*Agree*	*Strongly Agree*
1	*2*	*3*	*4*	*5*

17. I benefit by wearing personal protective equipment.

Strongly Disagree	*Disagree*	*Neither Agree or Disagree*	*Agree*	*Strongly Agree*
1	*2*	*3*	*4*	*5*

PERCEIVED BARRIERS

18. Wearing personal protective equipment is uncomfortable.

Strongly Disagree	*Disagree*	*Neither Agree or Disagree*	*Agree*	*Strongly Agree*
1	*2*	*3*	*4*	*5*

19. Personal protective equipment interferes with my ability to do the job.

Strongly Disagree	*Disagree*	*Neither Agree or Disagree*	*Agree*	*Strongly Agree*
1	*2*	*3*	*4*	*5*

20. Personal equipment is not always available to me.

Strongly Disagree	*Disagree*	*Neither Agree or Disagree*	*Agree*	*Strongly Agree*

1	*2*	*3*	*4*	*5*

21. My coworkers would make fun of me for wearing personal protective equipment.

Strongly Disagree	*Disagree*	*Neither Agree or Disagree*	*Agree*	*Strongly Agree*
1	*2*	*3*	*4*	*5*

22. My supervisor seldom wears personal protective when required.

Strongly Disagree	*Disagree*	*Neither Agree or Disagree*	*Agree*	*Strongly Agree*
1	*2*	*3*	*4*	*5*

23. My supervisor is aware of my compliance with personal protective equipment guidelines.

Strongly Disagree	*Disagree*	*Neither Agree or Disagree*	*Agree*	*Strongly Agree*
1	*2*	*3*	*4*	*5*

24. I would need to develop a new habit for wearing personal protective equipment, and this is difficult.

Strongly Disagree	*Disagree*	*Neither Agree or Disagree*	*Agree*	*Strongly Agree*
1	*2*	*3*	*4*	*5*

25. Wearing personal protective equipment is just too inconvenient for me.

Strongly Disagree	*Disagree*	*Neither Agree or Disagree*	*Agree*	*Strongly Agree*
1	*2*	*3*	*4*	*5*

<u>CUES TO ACTION</u>

26. A reminder from my supervisor every day would be important to my wear of personal protective equipment.

Strongly Disagree	*Disagree*	*Neither Agree or Disagree*	*Agree*	*Strongly Agree*
1	*2*	*3*	*4*	*5*

27. My supervisor checking on me would improve my wear of personal protective equipment.

Strongly Disagree	*Disagree*	*Neither Agree or Disagree*	*Agree*	*Strongly Agree*
1	*2*	*3*	*4*	*5*

28. The fact that OSHA could fine me or my employer for NOT wearing personal protective equipment is important.

Strongly Disagree	*Disagree*	*Neither Agree or Disagree*	*Agree*	*Strongly Agree*
1	*2*	*3*	*4*	*5*

29. Posters in my facility would serve as important reminders to wear personal protective equipment.

Strongly Disagree	*Disagree*	*Neither Agree or Disagree*	*Agree*	*Strongly Agree*
1	*2*	*3*	*4*	*5*

30. The threat of disciplinary action is an important factor in ensuring I wear personal protective equipment.

Strongly Disagree	*Disagree*	*Neither Agree or Disagree*	*Agree*	*Strongly Agree*
1	*2*	*3*	*4*	*5*

31. Having personal protective equipment at the location of the hazard is critical to making sure I wear it.

Strongly Disagree	*Disagree*	*Neither Agree or Disagree*	*Agree*	*Strongly Agree*
1	*2*	*3*	*4*	*5*

32. If I see others wearing personal protective equipment in my area, then it reminds me to use it.

Strongly Disagree	*Disagree*	*Neither Agree or Disagree*	*Agree*	*Strongly Agree*
1	*2*	*3*	*4*	*5*

33. Regular and frequent education on the importance of personal protective equipment serves to improve how often I wear it.

Strongly Disagree	*Disagree*	*Neither Agree or Disagree*	*Agree*	*Strongly Agree*
1	*2*	*3*	*4*	*5*

34. My supervisor sets the example on wearing personal protective equipment when exposed to hazards.

Strongly Disagree	*Disagree*	*Neither Agree or Disagree*	*Agree*	*Strongly Agree*
1	*2*	*3*	*4*	*5*

SELF-EFFICACY

35. I am confident that I will remember to use personal protective equipment when I am exposed to work hazards at work.

Strongly Disagree	*Disagree*	*Neither Agree or Disagree*	*Agree*	*Strongly Agree*
1	*2*	*3*	*4*	*5*

36. I am confident that I can obtain the proper personal protective equipment when I am exposed to hazards at work.

Strongly Disagree	*Disagree*	*Neither Agree or Disagree*	*Agree*	*Strongly Agree*
1	*2*	*3*	*4*	*5*

37. I am confident that my job performance will NOT be impacted by wearing personal protective equipment.

Strongly Disagree	*Disagree*	*Neither Agree or Disagree*	*Agree*	*Strongly Agree*
1	*2*	*3*	*4*	*5*

38. I am confident that the personal protective equipment I use when I am exposed to hazards at work is the proper equipment to protect me.

Strongly Disagree	*Disagree*	*Neither Agree or Disagree*	*Agree*	*Strongly Agree*
1	*2*	*3*	*4*	*5*

39. I am confident that after wearing the proper personal protective equipment throughout my career will prevent me from getting an occupational illness.

Strongly Disagree	*Disagree*	*Neither Agree or Disagree*	*Agree*	*Strongly Agree*
1	*2*	*3*	*4*	*5*

SECTION III: MANAGEMENT QUESTIONS

These questions pertain only to SUPERVISORS & MANAGERS at this facility. If you are NOT a SUPERVISOR OR MANAGER, then please skip to SECTION IV below. If you are a SUPERVISOR OR MANAGER, then please circle the number that corresponds most closely to the extent of your level of agreement with each statement.

40. How often do you enforce wearing personal protective equipment?

Never	*Rarely*	*Sometimes*	*Very Often*	*Always*
1	2	3	4	5

41. How often do you set the example on wearing personal protective equipment when being exposed to hazards?

Never	*Rarely*	*Sometimes*	*Very Often*	*Always*
1	2	3	4	5

42. How often are you aware of your employees' compliance to personal protective equipment?

Never	*Rarely*	*Sometimes*	*Very Often*	*Always*
1	2	3	4	5

43. How often do you threaten disciplinary action if personal protection equipment regulations are not followed?

Never	*Rarely*	*Sometimes*	*Very Often*	*Always*
1	2	3	4	5

44. How often do you ensure that personal protective equipment is available for your employees?

Never	*Rarely*	*Sometimes*	*Very Often*	*Always*
1	2	3	4	5

45. How often do you provide regular and frequent education on the importance of personal protective equipment?

Never	*Rarely*	*Sometimes*	*Very Often*	*Always*
1	*2*	*3*	*4*	*5*

SECTION IV: GENERAL EMPLOYEE INFORMATION

Please circle all that applies, and as with the previous conditions, everything will remain confidential.

Current age:

18-25 26-30 31-35 36-40 41-45 46-50 51-55 56-60 >60

Gender: Male Female Other ________________________

Total time in the wastewater industry:

Less than year 1-5 years 6-10 years 11-15 years 16-20 years over 20 years

What type of training on Personal Protective Equipment have you done during your career? (circle all that apply)

Familiarization training

Basic safety training

Hazard Communication Training

Supervisor safety training

Advanced personal protective training (OSHA courses, manufacturer courses, etc.)

THANK YOU FOR YOUR TIME!

Table S1. Occupation of Participants

Characteristics	Frequency (n)	Percent (%)
**Occupation**		
Accounts Coordinator	2	0.7
Admin Specialist	1	0.4
Biosolids Coordinator	1	0.4
Crew Leader	5	1.8
Crew Supervisor	2	0.7
Crew Worker	3	1.1
Dryer Technician	2	0.7
Engineer	5	1.8
Environmental Compliance Specialist	11	4.0
Field Support Technician	1	0.4
Heavy Equipment Operator	7	2.6
Industrial Pretreatment Technician	2	0.7
Instrument Technician	3	1.1
Lab Analyst/Chemist	14	5.1
Laboratory Supervisor	5	1.8
Locator	3	1.1
Maintenance Superintendent	8	2.9
Maintenance Supervisor	4	1.5
Maintenance Worker	34	12.5
Meter Technician	1	0.4
Natural Gas Technician	1	0.4
Safety Manager	2	0.7
Stormwater Technician	1	0.4
Unidentified	8	2.9
Utility Service Worker	1	0.4
Wastewater Collections Manager	3	1.1
Wastewater Collections Supervisor	4	1.5
Wastewater Collections Worker	9	3.3
Wastewater Operator	69	25.4
Wastewater Manager	20	7.4
Wastewater Superintendent	23	8.5
Wastewater Supervisor	17	6.3

Percentages based on completed response; This question involved multiple responses; the total responses will not equal 100%; The known job titles of the managers and supervisors include: Crew Leader, Crew Supervisor, Laboratory Supervisor, Maintenance Superintendent, Maintenance Supervisor, Safety Manager, Wastewater Collections Manager, Wastewater Collections Supervisor, Wastewater Manager, Wastewater Superintendent, and Wastewater Supervisor (n = 93); Additional participants who identified as supervisors and managers are unknown.

Table S2. Wastewater Licenses of Participants

Characteristics	Frequency (n)	Percent (%)
**Wastewater Licenses[a]**		
No Licenses or None Listed	90	29.4
Class 1 or Class A License	51	26.4
Class 2 or Class B License	18	8.0
Class 3 or Class C License	43	16.6
Class 4 or Class D License	24	6.6
Wastewater Collections License	49	16.4
Wastewater Lab Analyst License	18	8.9
Class 3 Maintenance Technologist	4	1.4
Master Electrician	1	0.3
Land Application	1	0.3

[a] This question involved multiple responses; the total responses will not equal 100%; Participants indicated all the wastewater licenses that they have earned while working in the wastewater industry.
